# Mixed thyrotropin-secreting pituitary neuroendocrine tumor coexisting with Graves' disease: a case report

**DOI:** 10.3389/fmed.2024.1436400

**Published:** 2024-09-04

**Authors:** Yijing Huang, Xiaoming Wen, Xinxin Liang, Lingling Xu

**Affiliations:** ^1^Department of Endocrinology, Shenzhen Hospital, Southern Medical University, Shenzhen, China; ^2^The Third School of Clinical Medicine, Southern Medical University, Guangzhou, China

**Keywords:** thyrotropin-secreting pituitary neuroendocrine tumor, mixed pituitary neuroendocrine tumor, Graves' disease, thyroid antibodies, autoimmune thyroid disease

## Abstract

**Background:**

Thyrotropin (TSH)-secreting pituitary neuroendocrine tumors (PitNETs) are recognized as a rare disease. Mixed TSH PitNETs account for 20–25% of TSH PitNETs. This study aimed to report an extremely rare case of a mixed TSH PitNET coexisting with Graves' disease (GD) and also to review the literature.

**Case presentation:**

A 36-year-old male patient presented with elevated levels of free triiodothyronine (FT3), free thyroxine (FT4), and insulin-like growth factor 1 (IGF-1) but a non-suppressed thyroid-stimulating hormone (TSH) level. His anti-thyroglobulin antibody (TgAb), anti-thyroid peroxidase autoantibody (TPOAb), and thyrotropin receptor antibody (TRAb) tests were positive. Symptoms of palpitations, hyperhidrosis, heat intolerance, and irritability appeared 2 years before his admission. However, he showed neither any signs nor any symptoms of acromegaly. The contrast-enhanced pituitary magnetic resonance imaging (MRI) showed enlargement of the pituitary fossa, with an irregular abnormal signal mass. The patient underwent endoscopic pituitary tumor resection via a transsphenoidal approach. The postoperative pathology suggested a mixed pituitary adenoma. At 8 months after the surgery, the patient had a postoperative recurrence of hyperthyroidism, and methimazole (MMI) was then administered. The recurrence of the TSH PitNET was confirmed by the positron emission tomography-computed tomography (PET-CT), which was performed 11 months after the surgery, and treatment with lanreotide was initiated. Gradually, his levels of FT3, FT4, TSH, TPOAb, and TgAb became normal and the levels of TRAb and IGF-1 improved.

**Conclusion:**

When the circulating levels of both FT4 and FT3 were upregulated, non-suppressed TSH levels and positive thyroid antibodies were found. TSH PitNETs coexisting with GD should be carefully taken into account to avoid the potential risk of treatment-induced tumor progression.

## Introduction

Adenohypophyseal tumors were renamed pituitary neuroendocrine tumors (PitNET) in 2020. Thyrotropin-secreting pituitary neuroendocrine tumors (TSH PitNETs) account for ~0.5–3% of all PitNETs. The incidence of TSH PitNETs is ~1/1,000,000, indicating its rarity. Up to 70–80% of TSH PitNETs secrete only TSH. In addition, 20–25% of TSH PitNETs are mixed PitNETs, which are characterized by concomitant hypersecretion of other anterior pituitary hormones, mainly including growth hormone (GH) or prolactin (PRL) (1). Graves' disease (GD), which is an autoimmune thyroid disease, is the most common cause of hyperthyroidism. TSH PitNETs are the main cause of central hyperthyroidism that can be easily misdiagnosed, especially when coexisting with GD. This may affect other hypophysis hormones, and even pituitary apoplexy, due to the enlarged TSH PitNET. Therefore, to avoid misdiagnosis, it is necessary to summarize the experience involving the diagnosis and treatment of the coexistence of the two diseases. Hence, the present study aimed to report an extremely rare case of a mixed TSH PitNET coexisting with GD.

## Case presentation

A 36-year-old Chinese male patient, who was admitted to our hospital on 21 August 2021, was included in this study. The patient's main complaints were rapid heartbeat and heat intolerance for 2 years, and a mass was found in his pituitary gland 1 month before his admission. The symptoms of palpitations, hyperhidrosis, heat intolerance, and irritability appeared 2 years before his admission, but he ignored them. Then, the patient suffered a femoral head fracture after trauma in 2020. After that, he underwent dual-energy X-ray absorptiometry (DXA). The total lumbar spine Z-scores were less than−2.0, which was diagnostic of osteoporosis. He was referred to another hospital due to osteoporosis 1 month before his admission. In that period, he was informed that his thyroid function was abnormal and his thyroid antibodies [thyroid peroxidase autoantibody (TPOAb), thyroglobulin antibody (TgAb), and thyrotropin receptor antibody (TRAb)] were positive. More importantly, when he was treated in another hospital, a mass was found in his pituitary gland, thus he came to our hospital to seek further diagnosis and treatment. His history of other diseases, receiving medications, and recent weight loss were negative. He denied a family history of thyroid disease.

During the physical examination at admission, the patient was 176 cm tall and weighed 83 kg (body mass index of 26.79 kg/m^2^). His blood pressure was 139/90 mmHg, and his heart rate was 97 beats/min. He had no manifestations of proptosis, edema, or enlargement of the thyroid.

**Figure 1:**
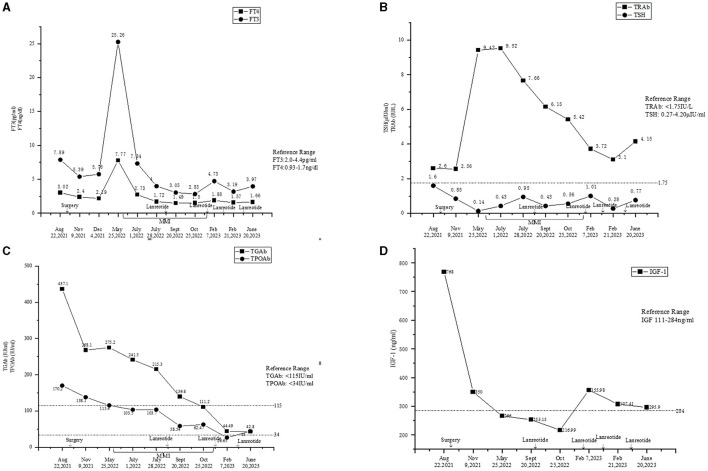
**(A)** Fluctuations of the levels of FT3 and FT4. **(B)** Fluctuations of the levels of TSH and TRAb. **(C)** Fluctuations of the levels of TgAb and TPOAb. **(D)** Fluctuations of the level of IGF-1.

**Figure 2:**
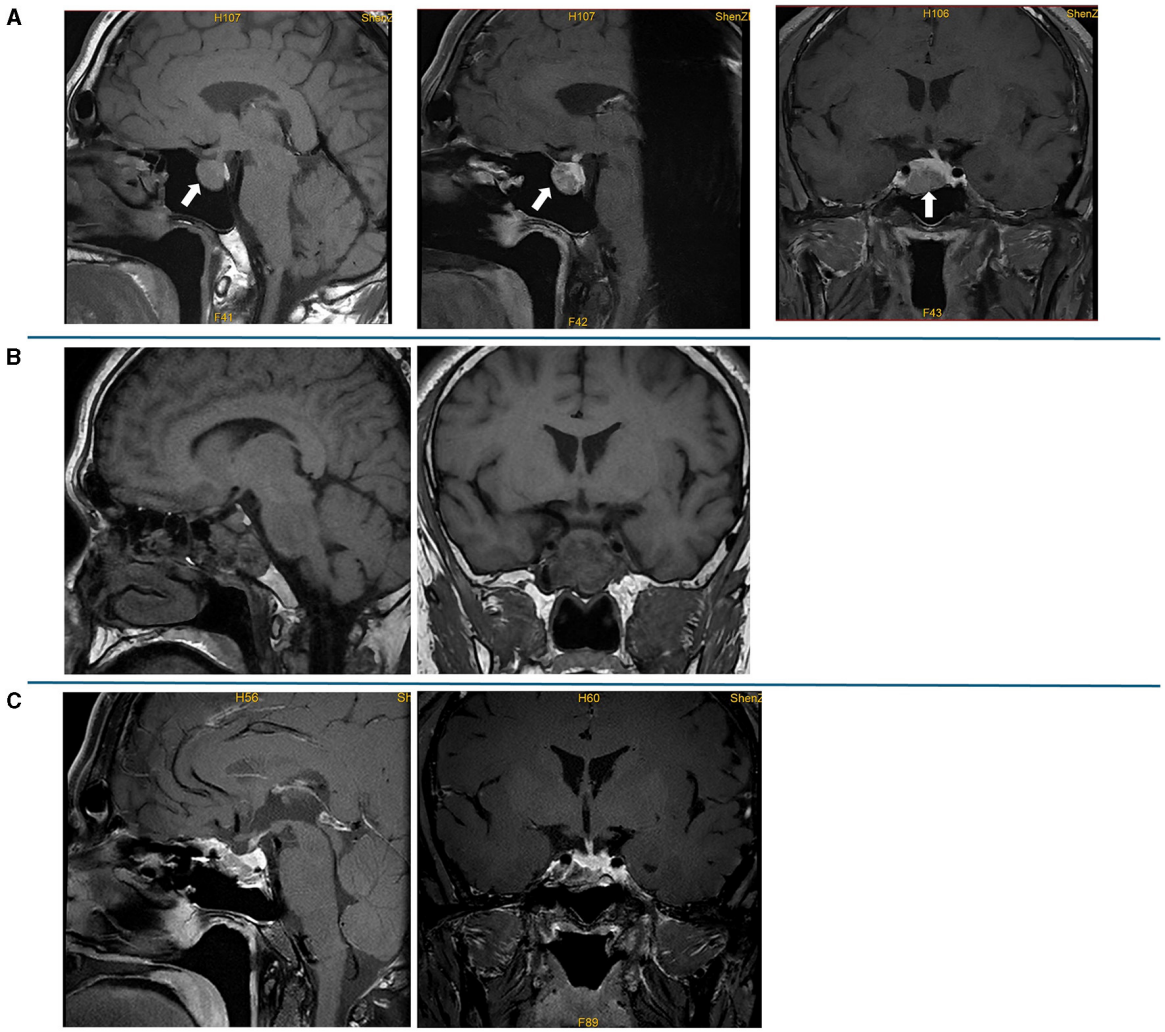
Preoperative and postoperative pituitary MRI. **(A)** Preoperative plain scan and contrast-enhanced sagittal MRI and contrast-enhanced sagittal and coronal MRI; a lesion (19 mm × 19 mm × 15 mm) in the pituitary fossa is indicated by an arrow. **(B)** Contrast-enhanced sagittal and coronal MRI on the first day after the surgery; the MRI showed postoperative changes in the pituitary gland, and sphenoid hemorrhagic sinus effusion was considered. **(C)** Contrast-enhanced sagittal and coronal MRI at 3 months after the surgery, which showed no mass lesion in the sella turcica.

The laboratory tests performed in our hospital showed that his blood, urine, fecal, liver and kidney functions, blood lipids, blood electrolytes, and urine electrolytes were not significantly abnormal. The thyroid function (Figures 1A, B) and thyroid antibodies (Figures 1B, C) were abnormal. Elevated levels of insulin-like growth factor 1 (IGF-1) and procollagen type I N-terminal propeptide (P1NP) were found, while the levels of GH, sex hormones, and sex hormone-binding globulin (SHBG) were in the normal range (Table 1). The blood cortisol and adrenocorticotropic hormone (ACTH) levels were in the normal range (Table 1). The chest X-ray findings were normal. The ultrasound showed heterogeneous echogenicity of the thyroid gland. The thyroid emission computed tomography (ECT) showed a slightly enlarged thyroid, which was normal in shape and position, and the distribution of the imaging agent was non-uniform. No obvious concentrated, sparse, or defective images of the localized imaging agents were found in the thyroid gland. The contrast-enhanced MRI of the pituitary gland showed enlargement of the pituitary fossa, with a mass (19 mm × 19 mm × 15 mm) of irregular abnormal signals within the region; the lesion grew from the sellar region to the suprasellar and parasellar regions, with depression in the sellar floor. The optic chiasm was compressed with mild elevation, with an unclear structure of the pituitary stalk and involvement of the right cavernous sinus; and it was in contact with the inner edge of the right internal carotid artery, without obvious wrapping. This indicated a macroadenoma (Figure 2A). The ophthalmic visual field examination showed no obvious abnormality.

**Table 1:** The results of the laboratory tests before and after the surgery.

**Laboratory test**	**22 August 2021 (before surgery)**	**9 November 2021 (1 month after surgery)**	**21 February 2023**	**Reference range**
GH	0.75	<0.05	1.4	0.06–5.0 ng/ml
IGF-1	768	350	307.41	109–284 ng/ml
FSH	6.01	5.34	5.97	1.5–12.4 mIU/ml
LH	2.27	1.96	2.76	1.7–8.6 mIU/ml
E2	33.45	27.75	17.4	11.3–43.2 pg/ml
PRL	7.67	8.25	8.48	4.04–15.2 ng/ml
Testosterone	8.89	5.27	3.58	2.49–8.36 ng/ml
Cortisol (8 am)	172.69	322.28	414.32	7am−9am 117.75–686.85 nmol/L
ACTH (8 am)	12.43	26.51	18.89	7am−9am 7.20–63.40 pg/ml
SHBG	43.48	33.18	35.34	14.55–94.64 nmol/L
P1NP	88	86.3	27.8	9.06–76.24 ng/ml

GH, Growth hormone; IGF-1, Insulin-like growth factor 1; FSH, Follicle-stimulating hormone; LH, Luteinizing hormone; E2, Estradiol; PRL, Prolactin; ACTH, Adrenocorticotropic hormone; SHBG, Sex hormone binding globulin; P1NP, Procollagen type I N-terminal propeptide.

For preoperative examination and surgery, the patient was transferred to Guangdong General Hospital (Guangzhou, China). A short-term somatostatin analog test was conducted to differentiate the TSH PitNET from resistance to thyroid hormone (RTH). Octreotide acetate was administered at a dose of 0.1 mg every 8 h. The serum TSH level was measured at 0, 2, 4, 6, 8, and 24 h after the first injection. The suppression rate of TSH at 24 h vs. 0 and 2 h was 73% and 62%, respectively. The suppression rate of TSH at 24 h vs. 2 h in this patient during hospitalization was 62%, which suggested a TSH PitNET. Genetic analysis detected no mutation in the thyroid hormone receptor (THR)-α or -β gene. He was diagnosed with a TSH PitNET.

**Figure 3:**
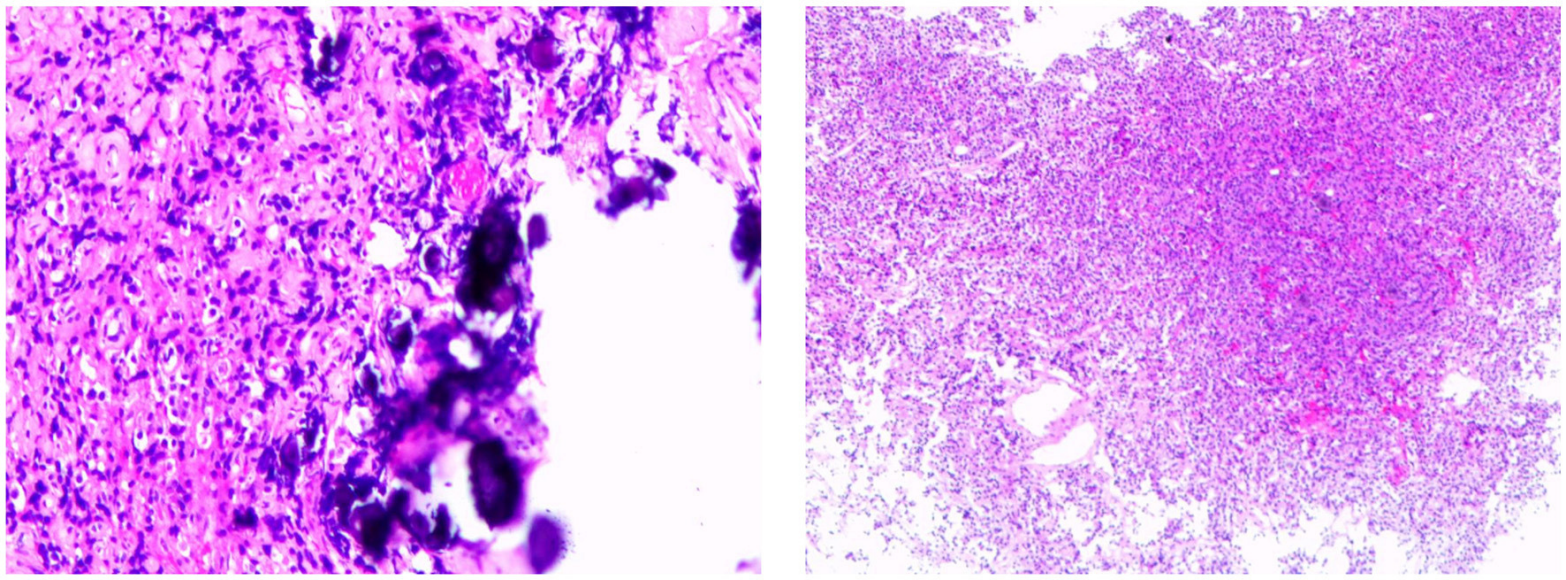
Postoperative pathology.

Octreotide acetate was administered (0.1 mg q12h) preoperatively. The patient underwent endoscopic pituitary tumor resection via transsphenoidal approach in Guangdong General Hospital on 22 September 2021. The postoperative contrast-enhanced pituitary MRI showed postoperative changes in the pituitary gland, and sphenoid hemorrhagic sinus effusion was considered (Figure 2B). The postoperative pathology suggested a pituitary adenoma (Figure 3). The immunohistochemical results were summarized as follows: ACTH (-), FSH (-), TSH (partial +), hGH (diffuse +), LH (-), PRL (weak +), Ki67 (<1%+), and CK (punctured +), which suggested that a mixed TSH PitNET coexisting with GD.

The levels of free triiodothyronine (FT3) and free thyroxine (FT4) at 1 and 2 months after the surgery reduced; however, they were still in abnormal ranges (Figure 1A). The thyroid antibodies were still positive at 1 month after the surgery (Figures 1B, C). The levels of IGF-1 (Figure 1D) and SHBG (Table 1) were downregulated, and the P1NP level reduced to 86.30 ng/ml (9.06~76.24 ng/ml), 1 month after the surgery. The levels of sex hormones, cortisol, and ACTH at follow-up were normal. The MRI showed no mass lesion in the sella turcica at 3 (Figure 2C), 8, and 11 months after the surgery.

**Figure 4:**
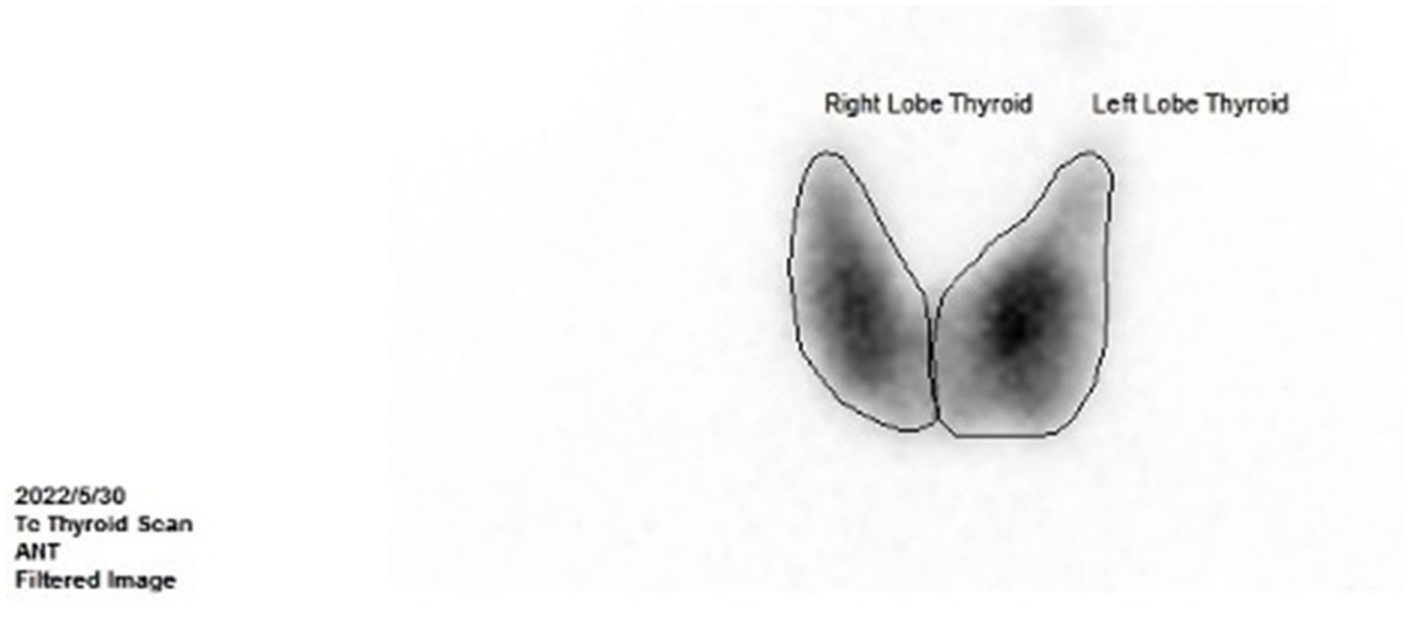
Thyroid ECT at 8 months after the surgery (showed moderate enlargement of the thyroid gland and an increase in the uptake of radionuclides Tc-99).

However, the patient complained of recurrence of palpitations, hyperhidrosis, and emaciation at 8 months after the surgery. The levels of FT3 and FT4 were elevated, while the TSH level was reduced (Figures 1A, B). The levels of TRAb (Figure 1B) and SHBG (126.29 nmol/L, reference range: 14.55–94.64 nmol/L) were significantly upregulated. The thyroid ECT showed moderate enlargement of the thyroid gland and an increase in the uptake of radionuclides Tc-99 (Figure 4). These results indicated hyperthyroidism. Methimazole (MMI) (10 mg) was administered orally twice per day starting from 29 May 2022. The levels of FT3 and FT4 decreased; however, they were still above the normal ranges. The TSH level was not suppressed (Figures 1A, B), and the levels of thyroid antibodies were still high 1 month later. The positron emission tomography-computed tomography (PET-CT) showed a nodular lesion at the right inferior margin of the sellar region with increased glucose metabolism, slightly increased amino acid metabolism, and positive somatostatin receptor imaging at 11 months after the surgery. The results of the PET-CT and the FT3, FT4, and TSH levels indicated the recurrence of the TSH PitNET. A single dose of lanreotide (90 mg) was administered on 21 August 2022. During the follow-up in September 2022 and October 2022, the levels of FT3, FT4, and TSH became normal. The MMI dose was subsequently and slowly reduced from 10 mg twice per day to 2.5 mg once per day and was discontinued on 3 January 2023. He received a second dose of lanreotide (90 mg) on 3 January 2023, more than 4 months after the first administration due to the COVID-19 epidemic prevention. The levels of FT3 and FT4 were slightly higher than normal ranges (Figure 1A), while the TRAb level significantly decreased (Figure 1B). The levels of TPOAb and TgAb were in the normal ranges (Figure 1C) on 7 February 2023. He then received a third dose of lanreotide (120 mg) on 14 February 2023. The IGF-1 level improved after the administration of lanreotide (Figure 1D). He then received lanreotide (120 mg) every 2 months starting from 14 February 2023. The follow-up hormonal and thyroid antibody results are shown in Figures 1A–D.

## Discussion and conclusion

The concurrence of GD and TSH PitNETs is rarely reported. According to the data retrieved from the PubMed database, there are only a few cases of pathologically confirmed TSH PitNETs coexisting with GD (2–9). The diagnosis requires differentiation between TSH PitNETs and RTH. Patients with TSH PitNETs usually have no family histories, while patients with RTH often have family histories. Adenomas are often found in patients with TSH PitNETs, while patients with RTH have no abnormal pituitary findings in imaging examinations. The measurement of serum glycoprotein hormone alpha-subunit (α-GSU) shows high circulating levels in ~70% of patients with TSH PitNETs (1), while the levels of serum α-GSU are reduced in patients with RTH. However, a measurement of α-GSU could not be carried out in the hospital. In addition, the methods and results of the octreotide inhibition test were not standardized. According to the literature review, the suppression rate of TSH at 24 h vs. 2 h was significantly greater in patients with TSH PitNETs compared to patients with RTH. The best diagnostic cut-off point of the suppression rate was 44.46% (10). Mutations in the gene-encoding thyroid hormone receptor β (THRβ) are most valuable for diagnosing RTH. This patient was diagnosed with a TSH PitNET and GD, but he was overweight, which may be related to the fact that the patient's hyperthyroidism was not severe or that the patient had a heavy baseline weight. His levels of FT3, FT4, and thyroid antibodies reduced after the surgery. However, the patient had a postoperative recurrence of hyperthyroidism, and the recurrence of the TSH PitNET was confirmed by PET-CT. After the lanreotide and MMI treatment, the patient's thyroid function gradually improved, the levels of the thyroid antibodies (TPOAb and TgAb) gradually decreased to normal ranges, and the TRAb level gradually reduced. This indicated that the patient's GD might have been related to the TSH PitNET or MMI might have had a delayed process of the effects, thus the exact cause of the postoperative improvement in the thyroid function and thyroid antibodies remains uncertain. Follow-up still needs to be continued after discontinuing MMI. Arai et al. (2) reported a 40-year-old woman diagnosed with a TSH PitNET along with GD. Her TSH PitNET was cured without recurrence, and her GD gradually improved postoperatively. Her TRAb levels decreased, and the TgAb level became negative. In another study (7), the patient was also diagnosed with a TSH PitNET complicated with GD, while his thyroid antibodies remained positive after pituitary surgery. His serum thyroid hormone level increased beyond the normal range 1 year after the surgery. In addition, the pituitary MRI showed a possible mass in the pituitary 3 years after the surgery, suggesting tumor recurrence. These two cases also indicated that their GD might be related to TSH PitNETs. One explanation for these findings is that abnormal hypersecretion of TSH may produce anti-idiotypic antibodies, causing GD, and normalization of TSH secretion by treating TSH PitNETs may reduce the production of these antibodies, thereby improving GD (4).

Some reports suggest that GD occurs after the surgical treatment of TSH PitNETs as thyroid autoantibodies tend to be elevated after pituitary surgery. According to the condition of our patient, the level of TRAb also elevated after surgery. Fas antigen is functionally expressed on the surface of thyroid cells, and TSH inhibits Fas antigen-mediated apoptosis of thyroid cells and promotes thyroid growth. Intercellular adhesion molecule-1 (ICAM-1) and major histocompatibility complex (MHC) II molecules are thought to be causative factors in autoimmune thyroid disease, and their expressions can be suppressed by TSH. Thus, a rapid decrease in TSH levels after surgery may induce apoptosis and activate autoimmune responses against the thyroid by increasing the expression of Fas, ICAM-1, and MHC II molecules on the thyroid cell surface (11).

The present case was a young male patient with osteoporosis. Osteoporosis associated with hyperthyroidism is mainly regarded as a secondary consequence of altered thyroid function. TSH acts as a single molecular switch in the independent control of both bone formation and resorption. Thus, TSH can inhibit osteoclast formation and survival by attenuating signals triggered in response to tumor necrosis factor-α (TNF-α). TSH also suppresses osteoblast differentiation and type I collagen expression (12).

In the present study, the patient showed neither signs nor symptoms of acromegaly. Although the basal GH level was normal with the elevated IGF-1 level, he was eventually found to have a mixed TSH PitNET that also secreted GH and PRL in the postoperative pathology. Unfortunately, our clinical analysis of the patient's preoperative clinical manifestations, as well as normal basal GH, and elevated IGF-1 was incomprehensive, thus the OGTT inhibition test of GH was not conducted. Multiple studies (13–15) have focused on GH-secreting PitNETs with an elevated IGF-1 level and a normal basal GH level. The hypothesis is that this is an entity with a distinct biological behavior characterized by a low GH level or is a classical acromegaly detected in its early stages (14). TSH/GH PitNETs tend to have a larger tumor diameter than the “solo-secreting TSH pituitary macro-neuroendocrine tumor,” and a high tendency of invasion is also found. The Ki-67 index is a biomarker of aggressive tumor behavior, which is related to the invasiveness, aggressiveness, and recurrence of a tumor (16). The present patient was diagnosed with a TSH/GH mixed PitNET that recurred postoperatively, which was consistent with previously reported findings.

According to existing guidelines and consensus, the first-line treatment for GH and TSH monosecreting PitNETs is surgery, which may not completely cure mixed GH/TSH PitNETs, on account of their fibrotic nature and invasiveness (17–19). In this sense, the choice of somatostatin analog injection as the initial treatment is conducive to rapid control of the oversecreted hormones and the efficacy of subsequent surgery. Indeed, comprehensive therapeutic regimens comprising two or three modalities were preferred in mixed GH/TSH PitNET cases in Yu et al.'s research (20). The patient's IGF-1 level reduced after the surgery and lanreotide treatment, indicating that the surgery and postoperative pharmacotherapy were effective. Few TSH PitNETs with low SHBG concentrations are those with concomitant hypersecretion of GH, which can potently inhibit SHBG secretion (21). This would explain the reason that the SHBG level was not elevated in the present case.

This study has some limitations. PitNETs were classified according to a combination of pituitary transcription factors (Pit-1, ER-3, SF-1, Tpit, etc.), hormones, and other biomarkers (keratin, Ki67, p27, FGFR4, etc.) (22). However, most of the pituitary transcription factors and biomarkers could not be carried out in our hospital. In addition, triiodothyronine (T3) suppression test, thyrotropin-releasing hormone (TRH) testing, and α-GSU were not available in our hospital. Moreover, the clinical laboratory of our hospital measured the total TRAb content in the patient's serum and could not measure the thyroid stimulating antibody (TSAb) alone. Finally, this review included some small-scale studies, such as case reports. Larger-scale clinical studies are needed to enhance our understanding of this rare disease.

In conclusion, a rare case of a TSH PitNET coexisting with GD was reported. When the circulating levels of both FT4 and FT3 were upregulated, non-suppressed TSH levels and positive thyroid antibodies were found. TSH PitNETs coexisting with GD should be carefully taken into account to avoid the potential risk of treatment-induced tumor progression.

## Data Availability

The datasets presented in this study can be found in online repositories. The names of the repository/repositories and accession number(s) can be found in the article/supplementary material.
